# Soil phosphorus cycling microbial functional genes of monoculture and mixed plantations of native tree species in subtropical China

**DOI:** 10.3389/fmicb.2024.1419645

**Published:** 2024-07-15

**Authors:** Lin Qin, Zhirou Xiao, Angang Ming, Jinqian Teng, Hao Zhu, Jiaqi Qin, Zeli Liang

**Affiliations:** ^1^Guangxi Key Laboratory of Forest Ecology and Conservation, College of Forestry, Guangxi University, Nanning, Guangxi, China; ^2^Experiment Center of Tropical Forestry, Chinese Academy of Forestry, Pingxiang, China; ^3^Guangxi Youyiguan Forest Ecosystem Research Station, Pingxiang, China

**Keywords:** planted forest, soil phosphorus cycling microbial functional genes, metagenomic sequencing, molecular ecological network structure, subtropical China

## Abstract

**Background:**

Transforming coniferous plantation into broadleaved or mixed broadleaved-coniferous plantations is the tendency of forest management strategies in subtropical China. However, the effects of this conversion on soil phosphorus (P) cycling microbial functional genes are still unknown.

**Methods:**

Soil samples were collected from 0–20, 20–40, and 40–60 cm (topsoil, middle layer, and subsoil, respectively) under coniferous *Pinus massoniana* (PM), broadleaved *Erythrophleum fordii* (EF), and their mixed (PM/EF) plantation in subtropical China. Used metagenomic sequencing to examine the alterations of relative abundances and molecular ecological network structure of soil P-cycling functional genes after the conversion of plantations.

**Results:**

The composition of P-cycling genes in the topsoil of PM stand was significantly different from that of PM/EF and EF stands (*p* < 0.05), and total phosphorus (TP) was the main factor causing this difference. After transforming PM plantation into EF plantation, the relative abundances of P solubilization and mineralization genes significantly increased in the topsoil and middle layer with the decrease of soil TP content. The abundances of P-starvation response regulation genes also significantly increased in the subsoil (*p* < 0.05), which may have been influenced by soil organic carbon (SOC). The dominant genes in all soil layers under three plantations were phoR, glpP, gcd, ppk, and ppx. Transforming PM into EF plantation apparently increased gcd abundance in the topsoil (*p* < 0.05), with TP and NO_3_^−^-N being the main influencing factors. After transforming PM into PM/EF plantations, the molecular ecological network structure of P-cycling genes was more complex; moreover, the key genes in the network were modified with the transformation of PM plantation.

**Conclusion:**

Transforming PM into EF plantation mainly improved the phosphate solubilizing potential of microorganisms at topsoil, while transforming PM into PM/EF plantation may have enhanced structural stability of microbial P-cycling genes react to environmental changes.

## 1 Introduction

Phosphorus (P) is a key macronutrient found in all living organisms and plays an essential role in plant growth and development (Reinhard et al., 2017). Although P is relatively abundant in soil, 95–99% of it is immobilized and thus can hardly be absorbed or utilized by plants (Lucero et al., 2021), making it a limiting nutrient for both soil microorganisms and plants (Wang et al., 2021). Phosphorus limitation in terrestrial ecosystems is considered a major issue that needs to be urgently addressed for ecosystem management and restoration (Liang et al., 2020).

Microorganisms exert important effects on soil P cycling and regulate its availability (Zaidi et al., 2009). The microbial P-transformation processes can be mostly regulated by 3 groups of microbial genes, namely, genes involved in inorganic P-solubilization and organic P-mineralization, P-uptake and transport, and P-starvation response regulation (Bergkemper et al., 2016). The genes related to P-starvation response regulation (*phoB*, *phoR*, and *phoU*) allow microorganisms to utilize P (Dai et al., 2020) and they also control the expression of genes related to organic P-mineralization (*phoD*) and P-uptake and transport (*pst*) (Hsieh and Wanner, 2010). Microorganisms have efficient P uptake systems, and thus, they compete with other soil biota for available P (Bergkemper et al., 2016; Wu et al., 2022). Genes encoding low-affinity inorganic phosphate transporter (*Pit*) and high-affinity phosphate transporter (*Pst*) play a significant role in promoting microbial phosphorus uptake and transport (Bergkemper et al., 2016). Microorganisms that carry genes related to inorganic P-solubilization and organic P-mineralization have been found to produce enzymes involved in mineralizing organic P or organic anions and solubilizing inorganic P (Dai et al., 2020). The Gene *gcd* is an important molecular biomarker for soil P-solubilizing microbes (Rawat et al., 2020), and *phoD* is a common gene responsible for mineralizing organic P (Hu et al., 2020) and is usually analyzed within the soil metagenome (Tan et al., 2012).

In recent years, soil microbial phosphorus cycling functional genes have attracted increasing attention, with the primary focus on agricultural ecosystems (Liu J. et al., 2018; Dai et al., 2020; Siles et al., 2022). Soil physicochemical properties are affected by various factors such as litter and root exudates from different vegetation types, and changes in their amounts drive the structure and functional responses of the soil microbial community (Bardgett and van der Putten, 2014). At the same time, soil physicochemical properties vary significantly with soil depth (Cade-Menun et al., 2017), causing alterations in soil microbial community abundance and structure at different soil depths (Becerra et al., 2014). Nonetheless, it remains largely unclear whether the abundance and structure of soil phosphorus cycling microbial functional genes vary among different stand types with soil layer. In addition, soil phosphorus cycling occurs as the result of an interaction between different microbial functional genes (Kelliher et al., 2018), and clarifying this interaction is the basis for understanding microbial community stability (Zhou et al., 2010). Molecular ecological network analysis used to characterize microbial functional genes provides a robust method to decipher the potential interactions of functional genes in complex microbial communities (Zhou et al., 2010; Liang et al., 2016). It has also been reported that the molecular ecological network structure of soil phosphorus cycling functional genes in natural evergreen-deciduous broadleaved mixed forests is more complex than that in natural evergreen broadleaved and deciduous broadleaved forests, indicating that soil phosphorus cycling microbial functional genes and their associated microbial communities in natural broadleaved mixed forests are more stable (Cheng et al., 2021). The key nodes in molecular ecological networks have an important and exclusive role in maintaining community stability, while the removal of these nodes from the networks dramatically changes microbial structure and functions (Banerjee et al., 2018). Therefore, identifying key microbial functional genes can shed light on the responses of microbial communities to environmental changes. However, the molecular ecological network structure of phosphorus cycling microbial functional genes in different planted forests is still unclear.

The establishment of native broadleaved plantations and coniferous-broadleaved mixed plantations is gradually becoming the most promising forest management approach to replace coniferous plantations distributed across large areas of the world (Liu S. et al., 2018; Wang et al., 2018) and also to improve soil fertility and forest productivity (Gillespie et al., 2021). Multiple generations of coniferous plantations can reduce litter decomposition rate, nutrient turnover rate, soil fertility, and net primary productivity (Schall and Ammer, 2012), while native broadleaved tree species have higher litter decomposition rate and soil fertility (You et al., 2018a), and the development of mixed-species plantations can promote soil nutrient turnover and improve woodland productivity (Handa et al., 2014). Phosphorus has a low availability in subtropical forest soils (You et al., 2020), and it is an important nutrient limiting plant development (Bergkemper et al., 2016). Therefore, understanding the effects of different planted forest management models on soil microbial phosphorus cycling functional genes can provide a reference for the selection of tree species and a scientific basis for the tree species distribution in subtropical regions. In this paper, the typical native coniferous *Pinus massoniana* (PM) plantation, broadleaved *Erythrophleum fordii* (EF) plantation, and PM/EF mixed plantation were studied in the subtropical region of China. Based on the metagenomic sequencing data of various soil depths (0–20, 20–40, and 40–60 cm) under all plantation stands, this study aims to investigate: (1) the alterations of the composition structure and relative abundance of soil microbial phosphorus cycling functional genes and the dominant soil physicochemical factors affected by transforming the coniferous plantation into mixed broadleaved - coniferous or broadleaved plantations; (2) the response profiles of the molecular ecological network structure of soil microbial phosphorus cycling functional genes to the conversion of the coniferous plantation to broadleaved or mixed broadleaved-coniferous plantations and also the potential keys genes in the network under three plantation types.

## 2 Materials and methods

### 2.1 Study site and sample collection

This experiment was conducted at the Daqingshan Experimental Field of the Experimental Center of Tropical Forestry, Chinese Academy of Forestry (22°10’ N,106°42′ E; with an altitude of 190–680 m above sea level), Pingxiang, Guangxi Zhuang Autonomy Region, China. Our study site is located in the subtropical region, with a typical subtropical monsoon climate and two distinguishable dry and wet seasons (the rainy season occurring from April to September and the dry season from October to March of the next year). The annual mean rainfall is 1,350 mm and relative humidity is above 80%. The landform types are mainly low mountains and hills, and the soil type is lateritic soil (Luo et al., 2014), which is the same as oxisol in the USDA Soil Taxonomy.

Three typical stands of PM, EF, and PM/EF were selected as research objects in this study (Table 1). These three stands were replanted on clear-cut sites of the PM stand in 2006, with a planting density of 2,500 plants/hm2 and 3:1 as the ratio of PM/EF stands. In January 2021, three sample plots (20 m × 20 m) were set up for each plantation, and plots were spaced at least 20 m apart to avoid spatial autocorrelation. In each sample plot, soil samples at the depths 0–20, 20–40, and 40–60 cm (topsoil, middle layer, and subsoil, respectively) (He et al., 2013; You et al., 2018b) were obtained using the soil drill (with an inner diameter of 5 cm) according to the 5-point “S-type” sampling method. Soil samples taken from the same layer were blended into one mixed sample. A total of 27 soil samples (3 stands × 3 plots × 3 soil layers) were obtained, placed into the sterile sampling bag, preserved on the biological ice pack, and quickly transferred to the laboratory. Each freshly collected mixed soil sample was divided into three parts, with one part being preserved in the refrigerator at −20°C following the filtration with the 2-mm sieve and then used for metagenomic sequencing. The second part was sieved with the 2-mm mesh and preserved in the refrigerator at 4°C to determine nitrate nitrogen (NO_3_^−^-N) and ammonium nitrogen (NH_4_^+^-N). The third part was sieved with the 0.25-mm mesh after natural air drying and then used for the determination of other soil physicochemical properties.

**Table 1:** The general information of the three studied planted forests.

Stand type	Altitude (m)	Aspect	Slope (°)	DBH (cm)	Tree height(m)	Density (tree/hm^2^)	Main understory vegetation
PM	190–240	NE	20	14.7	11.6	1700	*Microstegium vagans, Cyrtococcum patens, Lophatherum gracile*
EF	170–220	NE	25	13.7	13.7	1,125	*Lygodium japonicum, Miscanthus floridulus, Dicranopteris dichotoma*
PM/EF	180–230	NE	22	15.6	13.2	750	*Lygodium japonicum, Microstegium vagans, Cyrtococcum patens*

PM, *Pinus massoniana*; EF, *Erythrophleum fordii*.

### 2.2 Soil physicochemical parameters

We measured soil pH at the soil: water ratio of 1: 2.5 (w/v) with the pH meter (PHS-3C, Shanghai Jinhuan Instrument Co., Ltd., Shanghai, China). The potassium dichromate oxidation-external heating method was employed for determining soil organic carbon (SOC). The melt-molybdenum, antimony, and scandium colorimetry were used to measure total phosphorus (TP), whereas diacid (HCl-H_2_SO_4_) was used to extract available phosphorus (AP) which was then determined by enzymolysis (INFINITE M200 PRO, TECAN, Switzerland). The above-mentioned methods for the determination of soil physicochemical properties are described in detail by Bao (2000). Auto Analyzer (AutoAnalyzer3, SEAL, Germany) was used to measure total nitrogen (TN) content in the soil after de-boiling with H_2_SO_4_. KCl was used to extract NH_4_^+^-N and NO_3_^−^-N, which were then measured with Auto Analyzer (Wu et al., 2019). TOC analyzer (Multi N/C 3100, Analytik Jena AG, Germany) was used for measuring the dissolved organic carbon (DOC) content. The carbon-to-nitrogen ratio (C/N) in the soil is defined as the ratio of the mass of soil organic carbon to the mass of total nitrogen.

### 2.3 DNA isolation and metagenomic sequencing

The FastDNA^®^ Spin Kit for Soil was used for DNA extraction. DNA concentration, purity, and integrity were evaluated by electrophoresis on a 1% agarose gel. The genomic DNA was segmented into 400-bp fragments using the Covaris M220 instrument. The NEXTFLEX Rapid DNA-Seq Kit was adopted for paired-end (PE) library construction. The metagenomic sequencing was performed on the Illumina NovaSeq sequencing platform at Shanghai Meiji Biotechnology Co., Ltd. Besides, we applied the fastp tool v0.20.0 to quality control checks on original sequence data and optimize sequences for metagenomic analysis. Megahit software v1.1.2 was used to splice and assemble the optimized sequences, and overlapping groups of ≥300 bp from the splicing analysis were selected as the final assembly results for the next steps which were the gene prediction and annotation. MetaGene software was used to predict overlapping groups obtained from splicing analysis. We chose the genes with the nucleic acid length of ≥100 bp, translated them into amino-acid sequences, and finally obtained gene prediction statistics for every sample. Then, CD-HIT v4.6.1 was applied for clustering the gene sequences predicted in every sample (sequence similarity ≥90% and coverage ≥90%), while genes with the greatest length per class were chosen as the typical sequences for constructing the non-redundant gene set. SOAPaligner v2.21 was used to align high-quality sequences obtained from quality inspection of every sample and the non-redundant gene set (sequence similarity ≥95%). The abundance of each functional gene in the corresponding soil sample was calculated, and then the ratio of the abundance of each functional gene to the sum of the abundance of all functional genes in the sample was taken as the relative abundance of a single functional gene in the sample. The metagenomic sequencing data were imported into the National Microbiology Data Center^1^ (NMDC10018441).

Using BLASTP (BLAST Version 2.2.28+),^2^ sequences of redundant genes were compared with those of KEGG database,^3^ and the e-value of BLAST comparison parameters is set to 1e-5. According to the comparison results, KOBAS 2.0 (Xie et al., 2011) (KEGG Orthology Based Annotation System) was used for functional annotation. A total of 55 microbial phosphorus cycling functional genes obtained from all soil samples were grouped into three main categories (phosphorus-starvation response regulation, phosphorus absorption and transport, and inorganic phosphorus solubilization and organic phosphate mineralization) (Table 2).

**Table 2:** Classification of soil phosphorus cycling microbial functional genes.

Classification	KO number	Gene	Reference
**I. P*-*starvation response regulation**			
1. P*-*starvation response regulation			
(1) Phosphate regulon sensor histidine kinase	K07636	*phoR*	Choi et al. (2009) and Liu J. et al. (2018)
(2) Phosphate regulon response regulator	K07657	*phoB*	Choi et al. (2009) and Liu J. et al. (2018)
(3) Negative regulator of PhoR/PhoB two*-*component regulator	K02039	*phoU*	Choi et al. (2009) and Liu J. et al. (2018)
**II. P*-*uptake and transportation**			
2. Inorganic phosphate transportation			
(4) Phosphate transport system ATP*-*binding protein	K02036	*pstB*	Liu J. et al. (2018)
(5) Phosphate transport system permease protein	K02037	*pstC*	Liu J. et al. (2018)
(6) Phosphate transport system permease protein	K02038	*pstA*	Liu J. et al. (2018)
(7) Phosphate transport system substrate*-*binding protein	K02040	*pstS*	Liu J. et al. (2018)
(8) Inorganic phosphate transporter	K03306	*pit*	Liu J. et al. (2018)
3. Phosphate transportation			
(9) sn*-*glycerol 3*-*phosphate transport system substrate*-*binding protein	K05813	*ugpB*	Liang et al. (2020)
(10) sn*-*glycerol 3*-*phosphate transport system permease protein	K05814	*ugpA*	Liang et al. (2020)
(11) sn*-*glycerol 3*-*phosphate transport system permease protein	K05815	*ugpE*	Liang et al. (2020)
(12) sn*-*glycerol 3*-*phosphate transport system ATP*-*binding protein	K05816	*ugpC*	Liang et al. (2020)
(13) Glycerol uptake operon antiterminator	K02433	*glpP*	Beijer and Rutberg (1992)
(14) Glycerol*-*3*-*phosphate transporter	K02445	*glpT*	Beijer and Rutberg (1992)
4. Phosphonate transportation			
(15) Phosphonate transport system ATP*-*binding protein	K02041	*phnC*	Liu J. et al. (2018)
(16) Phosphonate transport system permease protein	K02042	*phnE*	Liu J. et al. (2018)
(17) Phosphonate transport system substrate*-*binding protein	K02044	*phnD*	Liu J. et al. (2018)
(18) 2*-*amino*-*ethyl phosphonate transport system permease protein	K11082	*phnV*	Jiang et al. (1995)
**III. Inorganic P*-*solubilization and organic P *–*mineralization**			
5. Inorganic phosphate solubilization			
(19) Quinoprotein glucose dehydrogenase gcd	K00117	*gcd*	Andreeva et al. (2011) and An and Moe (2016)
(20) Polyphosphate kinase	K00937	*ppk*	Liu J. et al. (2018)
(21) Inorganic pyrophosphatase	K01507	*ppa*	Liu J. et al. (2018)
(22) Exopolyphosphatase	K01524	*ppx*	Liu J. et al. (2018)
(23) B Pyrroloquinoline quinone biosynthesis protein B	K06136	*pqqB*	Andreeva et al. (2011) and An and Moe (2016)
(24) Pyrroloquinoline*-*quinone synthase	K06137	*pqqC*	Andreeva et al. (2011) and An and Moe (2016)
(25) Pyrroloquinoline quinone biosynthesis protein	K06138	*pqqD*	Andreeva et al. (2011) and An and Moe (2016)
(26) PqqA peptide cyclase	K06139	*pqqE*	Andreeva et al. (2011) and An and Moe (2016)
6. Phosphate ester mineralization			
(27) Alpha*-*glycerophosphate oxidase	K00105	*glpO*	Beijer and Rutberg (1992)
(28) Glycerol*-*3*-*phosphate dehydrogenase	K00111	*glpA*	Beijer and Rutberg (1992)
(29) Glycerol*-*3*-*phosphate dehydrogenase subunit B	K00112	*glpB*	Beijer and Rutberg (1992)
(30) Glycerol*-*3*-*phosphate dehydrogenase subunit C	K00113	*glpC*	Beijer and Rutberg (1992)
(31) Glycerol kinase	K00864	*glpK*	Beijer and Rutberg (1992)
(32) Glycerol uptake facilitator protein	K02440	*glpF*	Beijer and Rutberg (1992)
(33) Glycerol*-*3*-*phosphate regulon repressor	K02444	*glpR*	Beijer and Rutberg (1992)
(34) Alkaline phosphatase	K01077	*phoA*	Liu J. et al. (2018)
(35) Alkaline phosphatase D	K01113	*phoD*	Liu J. et al. (2018)
(36) Acid phosphatase	K01078	*olpA*	Liu J. et al. (2018)
(37)(A)Acid phosphatase (class A)	K09474	*phoN*	Liu J. et al. (2018)
(38) 3*-*phytase	K01083	*phyA*	Liu J. et al. (2018)
(39) 4*-*phytase/acid phosphatase	K01093	*appA*	Liu J. et al. (2018)
(40) Phosphotriesterase*-*related protein	K07048	*opd*	Beijer and Rutberg (1992)
(41) Glycerophosphoryl diester phosphodiesterase	K01126	*ugpQ*	Beijer and Rutberg (1992)
7. Phosphonate mineralization			
(42) C*-*P lyase core complex, Phosphonate transport system regulatory protein	K02043	*phnF*	Liu J. et al. (2018)
(43) C*-*P lyase core complex, Ribose 1,5*-*bisphosphokinase	K05774	*phnN*	Liu J. et al. (2018)
(44) C*-*P lyase core complex, Alpha*-*D*-*ribose 1*-*methylphosphonate 5*-*triphosphate synthase subunit	K05780	*phnL*	Liu J. et al. (2018)
(45) C*-*P lyase core complex, Alpha*-*D*-*ribose 1*-*methylphosphonate 5*-*triphosphate synthase subunit	K05781	*phnK*	Liu J. et al. (2018)
(46) C*-*P lyase core complex, Alpha*-*D*-*ribose 1*-*methylphosphonate 5*-*triphosphate diphosphatase	K06162	*phnM*	Liu J. et al. (2018)
(47) C*-*P lyase core complex, Alpha*-*D*-*ribose 1*-*methylphosphonate 5*-*triphosphate synthase subunit	K06163	*phnJ*	Liu J. et al. (2018)
(48) C*-*P lyase core complex, Alpha*-*D*-*ribose 1*-*methylphosphonate 5*-*triphosphate synthase subunit	K06164	*phnI*	Liu J. et al. (2018)
(49) C*-*P lyase core complex, Alpha*-*D*-*ribose 1*-*methylphosphonate 5*-*triphosphate synthase subunit	K06165	*phnH*	Liu J. et al. (2018)
(50) C*-*P lyase core complex, Alpha*-*D*-*ribose 1*-* methylphosphonate 5*-*triphosphate synthase subunit	K06166	*phnG*	Liu J. et al. (2018)
(51) C*-*P lyase core complex, Phosphoribosyl 1,2*-*cyclic phosphate phosphodiesterase	K06167	*phnP*	Liu J. et al. (2018)
(52) C*-*P lyase core complex, Aminoalkylphosphonate N*-*acetyltransferase	K09994	*phnO*	Liu J. et al. (2018)
(53) Alkylphosphonate utilization operon protein	K06193	*phnA*	Liu J. et al. (2018)
(54) 2*-*aminoethylphosphonate*-*pyruvate transaminase	K03430	*phnW*	Liu J. et al. (2018)
(55) Phosphonoacetaldehyde hydrolase	K05306	*phnX*	Liu J. et al. (2018)

### 2.4 Statistical analysis

Effects of stand type, soil layer, and their interaction on the relative abundances of phosphorus cycling function genes and soil physicochemical properties were determined by the two-way analysis of variance (ANOVA), and Duncan’s multiple range test (MRT) was used for multiple comparisons of different treatment groups. Before performing ANOVA, all data were subjected to Levene’s test for the homogeneity of variance and Shapiro -Wilk normality test, and data that did not conform to the assumptions of ANOVA were transformed to log10. The calculations were carried out by using SPSS 26.0 (IBM SPSS Inc., Chicago, IL, USA). According to the Bray-Curtis distance calculated from the relative abundances of phosphorus cycling functional genes in each soil sample, principal coordinate analysis (PCoA), permutational multivariate analysis of variance (PERMANOVA), and redundancy analysis (RDA) were performed to investigate the differences in the composition structure of soil phosphorus cycling microbial functional genes in different stands and the main soil physicochemical factors leading to these differences. Collinear variables whose variance inflation factor (VIF) was >10 were excluded from the RDA model (Liu J. et al., 2018). The calculation was performed by the “cmdscale ()” function, “adonis ()” function, and “dbrda ()” function of the “vegan” package in R software v 4.0.5 (Dixon, 2003).

Pearson’s correlation was employed to analyze the relationships between soil physicochemical properties and relative abundances of phosphorus cycling functional genes. The correlation heatmap was created by the “corr. Test ()” function of the psych package in R software v 4.0.5. Using the “lavaan” package in R software, we performed structural equation modeling (SEM) to quantify the effects of stand types on soil phosphorus cycling functional gene groups. Topological parameters of the molecular ecological network were calculated in the MENA platform^4^ for P-cycling microbial functional genes, and network visualization was carried out using Cytoscape 3.8.0 software.^5^ The nodes of each network were classified into four categories based on their topological roles: (1) peripherals (*Zi* < 2.5 and *Pi* < 0.62); (2) connectors (*Zi* < 2.5 and *Pi* ≥ 0.62); (3) module hubs (*Zi* ≥ 2.5 and *Pi* < 0.62); and (4) network hubs (*Zi* ≥ 2.5 and *Pi* ≥ 0.62). Among them, the last three are regarded as the key nodes in molecular ecological networks (Olesen et al., 2007).

## 3 Results

### 3.1 Soil properties

According to the two-way ANOVA, stand type only dramatically affected soil TP and TN, while soil layer remarkably affected SOC, DOC, TP, and C/N, and the interaction between stand type and soil layer apparently affected only soil TP (*p* < 0.05) (Table 3). The soil TP contents varied among PM, EF, and PM/EF stands with soil layer. In the topsoil (0–20 cm), the TP content of the PM stand remarkably increased compared to that of PM/EF and EF stands (*p* < 0.05). In the middle layer and subsoil (20–40 and 40–60 cm, respectively), the TP content of the PM stand had no significant difference from that of the PM/EF stand, but remarkably increased compared to that of the EF stand. In addition, TN and NO_3_^−^-N contents in the subsoil of the PM plantation were significantly lower than those of the PM/EF plantation. Differences in the other 6 soil physicochemical parameters, including pH, SOC, DOC, NO_3_^−^-N, AP, and C/N in all soil layers were not significant among the three plantation stands.

**Table 3:** Comparison of soil physicochemical properties among three different plantations (mean ± SD, *n =* 3).

Stand type	Soil layer	pH	SOC (g‧kg^−1^)	DOC (mg‧kg^−1^)	TN (g‧kg^−1^)	TP (g‧kg^−1^)	NO_3_^−^-N (mg‧kg^−1^)	NH_4_^+^-N (mg‧kg^−1^)	AP (mg‧kg^−1^)	C/N
PM	0–20 cm	4.56 ± 0.11aA	26.87 ± 2.32aA	17.67 ± 3.69aA	1.18 ± 0.18aA	0.40 ± 0.04aA	0.48 ± 0.25aA	22.42 ± 4.96aA	1.04 ± 0.03aA	22.99 ± 3.16aA
	20–40 cm	4.59 ± 0.20aA	21.14 ± 0.83aB	16.90 ± 0.07aA	1.31 ± 0.11aA	0.30 ± 0.02aB	0.42 ± 0.1aA	20.64 ± 1.61aA	0.73 ± 0.02aB	16.63 ± 2.05aB
	40–60 cm	4.57 ± 0.11aA	16.07 ± 2.39aC	15.47 ± 3.29aA	1.04 ± 0.15bA	0.37 ± 0.02aA	0.35 ± 0.37bA	16.71 ± 0.47aA	0.50 ± 0.02aC	15.76 ± 3.55aB
EF	0–20 cm	4.43 ± 0.08aA	23.04 ± 2.87aA	22.51 ± 11.12aA	1.22 ± 0.22aA	0.28 ± 0.02bA	1.56 ± 1.46aA	18.30 ± 3.90aA	0.76 ± 0.24aA	19.17 ± 3.81aA
	20–40 cm	4.42 ± 0.21aA	18.07 ± 3.02aAB	15.69 ± 3.26aA	1.06 ± 0.11aA	0.23 ± 0.03bA	0.64 ± 0.65aA	16.05 ± 2.04aA	0.50 ± 0.12aA	16.99 ± 2.26aA
	40–60 cm	4.48 ± 0.15aA	14.87 ± 3.35aB	10.79 ± 2.31aA	1.10 ± 0.22abA	0.28 ± 0.04bA	0.52 ± .47abA	17.47 ± 6.30aA	0.47 ± 0.16aA	14.31 ± 6.45aA
PM/EF	0–20 cm	4.50 ± 0.06aA	27.38 ± 4.12aA	21.30 ± 4.51aA	1.30 ± 0.17aA	0.33 ± 0.03bA	1.33 ± 1.03aA	24.03 ± 3.82aA	0.90 ± 0.20aA	21.36 ± 4.58aA
	20–40 cm	4.56 ± 0.06aA	21.97 ± 5.70aAB	17.17 ± 5.00aA	1.22 ± 0.20aA	0.36 ± 0.03aA	0.71 ± 0.52aA	21.64 ± 3.26aA	1.03 ± 1.00aA	18.89 ± 7.67aA
	40–60 cm	4.52 ± 0.05aA	17.13 ± 2.95aB	13.56 ± 6.45aA	1.43 ± 0.16aA	0.37 ± 0.05aA	1.20 ± 0.03aA	21.19 ± 5.09aA	0.71 ± 0.33aA	12.20 ± 3.43aA
Two*-*way ANOVA	Stand	ns	ns	ns	*	***	ns	ns	ns	ns
	Layer	ns	***	*	ns	*	ns	ns	ns	*
	Stand×Layer	ns	ns	ns	ns	*	ns	ns	ns	ns

PM, *Pinus massoniana*; EF, *Erythrophleum fordii*; SOC, Soil organic carbon; DOC, Dissolved organic carbon; TN, Total nitrogen; TP, Total phosphorus; NO_3_^−^-N, Nitrate nitrogen; NH_4_^+^-N, Ammonium nitrogen; and AP, Available phosphorus. Different lowercase letters indicate significant differences among different stand types for the same soil layer, whereas different uppercase letters denote significant differences among different soil layers for the same stand type at the 0.05 significance level.*p < 0.05; ***p < 0.001; and ns *p* > 0.05.

### 3.2 Composition structure of microbial genes involved in P transformation

**Figure 1:**
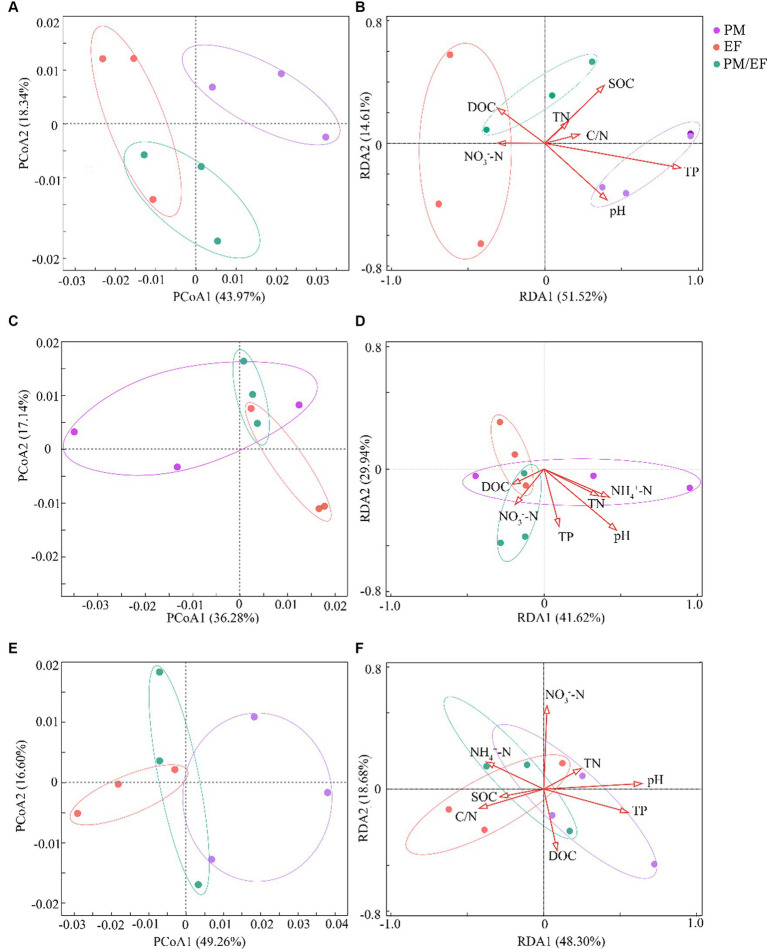
Principal coordinate analysis (PCoA) and redundancy analysis (RDA) of the composition structure of microbial phosphorus cycling functional genes in the three stands at different soil layers; **(A,B)** 0–20 cm; **(C,D)** 20–40 cm; and **(E,F)** 40–60 cm; PM, *Pinus massoniana*; EF, *Erythrophleum fordii.*

In the topsoil, principal coordinate analysis (PCoA) showed that the first axis (PCoA1) could explain 43.97% of the variation of the structure of soil phosphorus cycling microbial functional genes, while the second axis (PCoA2) accounted for 18.34% of this variation, and the total interpretation rate of the first two axes reached 62.31% (Figure 1A). The soil samples from the PM plantation were located on the right side of PCoA1, whereas the EF plantation soil samples were located on the left side of PCoA1, and the soil samples of the PM/EF plantation were concentrated below PCoA2. PERMANOVA analysis further demonstrated that the structure of soil phosphorus cycling genes in the PM plantation was significantly different from that in both EF and PM/EF plantations (*p* < 0.05). RDA analysis showed that the first two axes explained 66.13% (RDA1:51.52% and RDA2:14.61%) of the impact of soil physicochemical factors on the composition structure of phosphorus cycling functional genes (Figure 1B). Monte Carlo test revealed that soil TP was the main physicochemical factor that caused the significant difference in the composition structure of soil phosphorus cycling microbial functional genes among PM, EF, and PM/EF plantations (*p* < 0.05).

In the middle layer, the cumulative interpretation rate of the first two axes of PCoA was 53.42%, and the interpretation rates of PCoA1 and PCoA2 were 36.28 and 17.14%, respectively (Figure 1C); however, PERMANOVA analysis showed no significant difference among PM, EF, and PM/EF stands. According to the RDA analysis, the first two axes accounted for 41.62 and 29.94%, respectively, of the impacts of soil physical and chemical factors on the structure of soil phosphorus cycling microbial functional genes, explaining the total variation of 71.56% (Figure 1D). Monte Carlo test results showed that the differences in the structure of phosphorus cycling microbial genes among soil samples were not caused by the selected physical and chemical properties.

In the subsoil, however, the total interpretation rate of the first two axes of PCoA was 65.86% (PCoA1:49.26% and PCoA2:16.60%) (Figure 1E). Based on PERMANOVA results, PM, EF, and PM/EF plantation stands were not significantly different. Soil physical and chemical factors accumulated in the first two axes of RDA could explain 66.98% of variation in the composition structure of soil phosphorus cycling microbial functional genes (RDA1: 48.30% and RDA2: 18.68%) (Figure 1F). As revealed by Monte Carlo test results, soil pH were the major factors driving differences in the composition structure of phosphorus cycling functional genes in different soil samples (*p* < 0.05).

### 3.3 Relative abundances of genes involved in P transformation

#### 3.3.1 Categories of phosphorus cycling microbial functional genes

**Figure 2:**
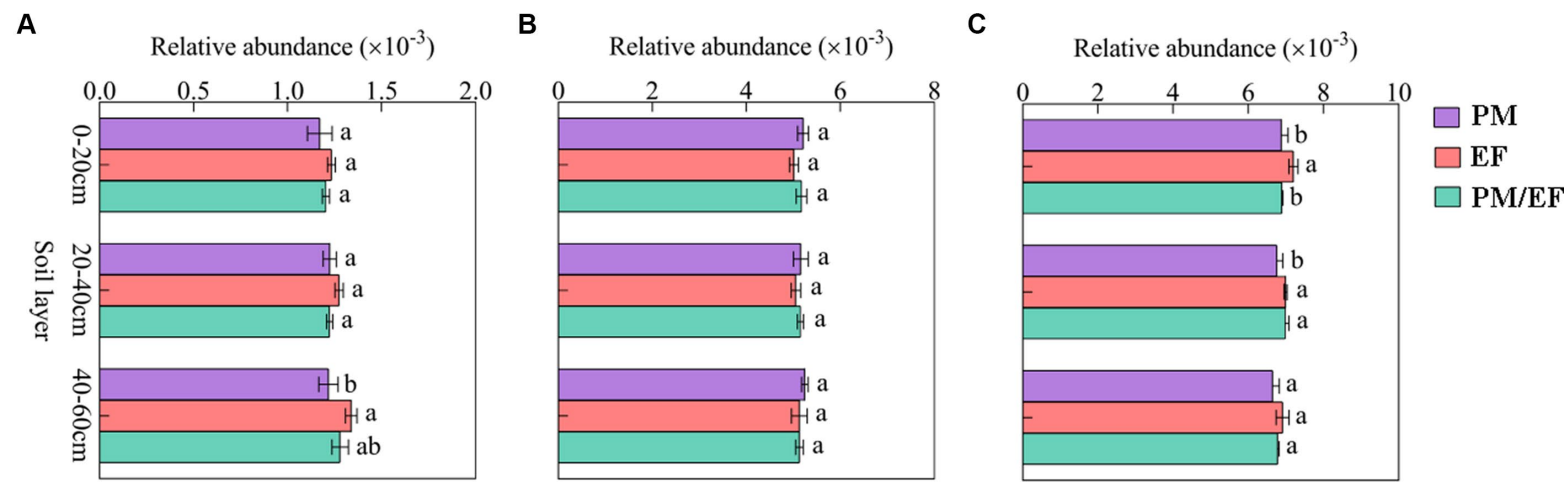
Relative abundances of the groups of phosphorus cycling microbial functional genes in different stands at different soil layers; **(A)** Genes related to P-starvation response regulation; **(B)** Genes related to P-uptake and transport; and **(C)** Genes related to P-solubilization and mineralization; PM, *Pinus massoniana*; EF, *Erythrophleum fordii.*

Based on the two-way ANOVA, stand types and soil layers dramatically affected the relative abundance of genes related to P-starvation response regulation, inorganic P-solubilization, and organic P-mineralization (*p* < 0.05), while relative abundances of genes involved in P-uptake and transportation were not affected by stand, soil layer, and their interaction (Table 4). The genes involved in P-starvation response regulation were not significantly different with regard to their relative abundances in the soil surface and middle layer, whereas, in the subsoil, the values remarkably decreased in the PM stand compared to those in the EF stand (*p* < 0.05) (Figure 2A). For genes involved in P-uptake and transportation, similarly, relative abundances did not show significant differences across three stands throughout the soil profile (Figure 2B). For genes involved in inorganic P-solubilization and organic P-mineralization, in contrast, relative abundances in the topsoil were significantly lower in the PM stand than in the EF stand. In the middle layer, the PM stand exhibited remarkably decreased values compared to EF and PM/EF stands, and no significant difference was observed among the three stands in the subsoil (Figure 2C).

**Table 4:** Results of the two*-*way ANOVA showing the impacts of stand types and soil layers on phosphorus cycling microbial functional genes.

Indicator	Source of variation	Degree of freedom	F	*P*
P-starvation response regulation	Stand	2	9.856	0.001**
	Layer	2	9.299	0.002**
	Stand × Layer	4	0.965	0.451
P-uptake and transport	Stand	2	3.539	0.051
	Layer	2	0.407	0.671
	Stand × Layer	4	0.493	0.741
P-solubilization and mineralization	Stand	2	10.406	0.001**
	Layer	2	6.324	0.008**
	Stand × Layer	4	1.146	0.367
*gcd*	Stand	2	11.649	0.001**
	Layer	2	13.268	0.000***
	Stand × Layer	4	1.666	0.201
*ugpQ*	Stand	2	3.202	0.065
	Layer	2	12.367	0.000***
	Stand × Layer	4	0.984	0.441
phnW	Stand	2	4.945	0.019*
	Layer	2	7.032	0.006**
	Stand × Layer	4	0.668	0.623
*phnX*	Stand	2	6.891	0.006**
	Layer	2	0.662	0.528
	Stand × Layer	4	1.334	0.296
*phoD*	Stand	2	1.708	0.209
	Layer	2	12.021	0.000***
	Stand × Layer	4	2.826	0.056
*phoR*	Stand	2	11.008	0.001**
	Layer	2	6.807	0.006**
	Stand × Layer	4	0.436	0.781

*p < 0.05; **p < 0.01; and ***p < 0.001.

**Figure 3:**
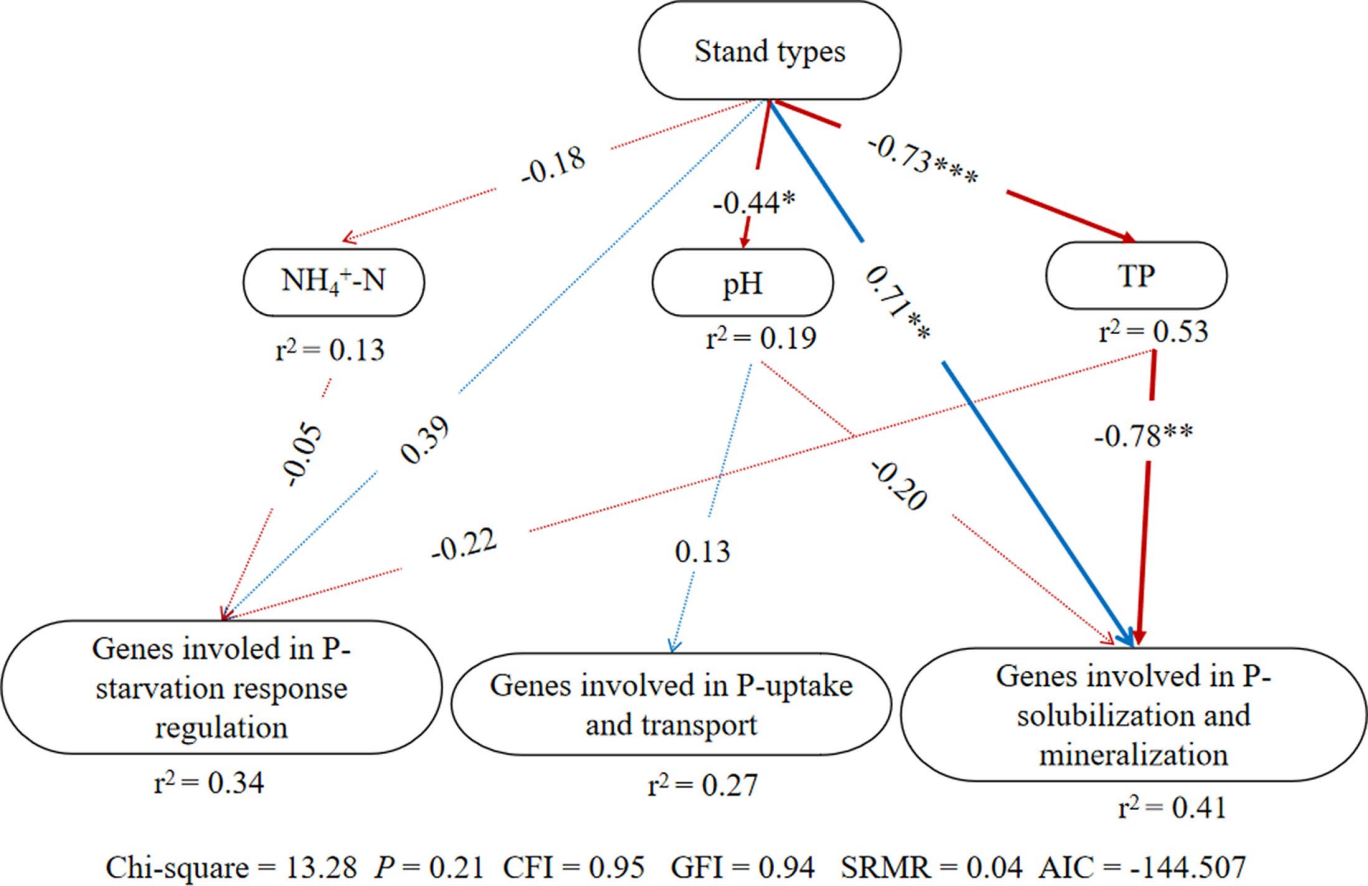
Structural equation model (SEM) illustrating the effects of stand types on groups of phosphorus cycling microbial functional genes; Arrow width indicates the strength of the relationship. Blue and red colors represent positive and negative relationships, respectively. Solid and dashed arrows represent significant and non-significant relationships among different variables, respectively (*p < 0.05, **p < 0.01, and ***p < 0.001). The values adjacent to arrows indicate path coefficients. r^2^: percentage of the variance in each dependent variable.

According to the structural equation modeling (SEM) results (Figure 3), *p*-value of Chi-square > 0.05, the comparative fit index (CFI) and goodness-of-fit index (GFI) were both >0.9, standardized root mean square residual (SRMR) < 0.09, indicating a good model fit (Hooper et al., 2008). There were no significant effects of stand types on genes involved in P-starvation response regulation and P-uptake and transportation, but they had direct significant positive effects on genes involved in inorganic P-solubilization and organic P-mineralization (*p* < 0.05). At the same time, stand types exerted indirect negative impacts on genes involved in inorganic P-solubilization and organic P-mineralization through the soil TP content.

#### 3.3.2 Species of phosphorus cycling microbial functional genes

**Figure 4:**
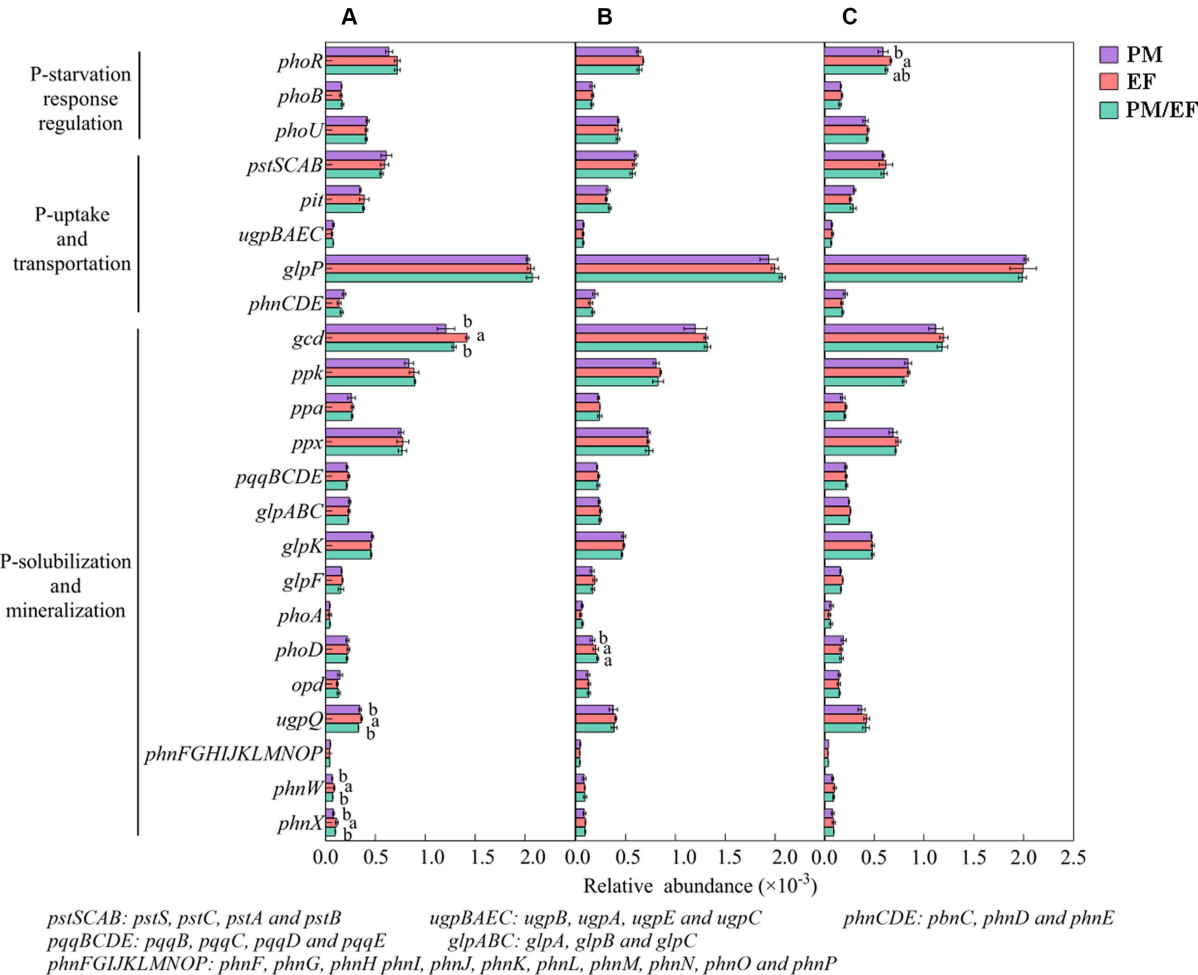
The relative abundance of phosphorus cycling microbial functional genes in three studied stands at soil layers of 0–20 **(A)**, 20–40 **(B)**, and 40–60 cm **(C)**; Functional genes with the relative abundance of less than 0.1‰ were eliminated. Different lowercase letters indicate significant differences among different stand types (*p* < 0.05).

Predominant functional genes in every soil layer for 3 stands were *phoR*, *glpP*, *gcd*, *ppk*, and *ppx*, with a relative abundance of >0.5‰ (Figure 4). The two-way ANOVA showed that stand types and soil layers remarkably affected the relative abundances of *phoR*, *gcd*, and *phnW* (*p* < 0.05), while *ugpQ* and *phoD* were significantly affected only by soil layers, and *phnX* was significantly affected only by stand types (Table 4). In addition, at the topsoil, the relative abundances of *gcd*, *ugpQ*, *phnW*, and *phnX* in the PM plantation apparently decreased compared to those in the EF plantation (*p* < 0.05) (Figure 4A); at the middle layer, similarly, the relative abundance of *phoD* in the PM plantation remarkably decreased compared with that of EF and PM/EF plantations (Figure 4B), and at the subsoil, the relative abundance of *phoR* in the PM plantation evidently decreased compared with that in the EF plantation (Figure 4C).

**Figure 5:**
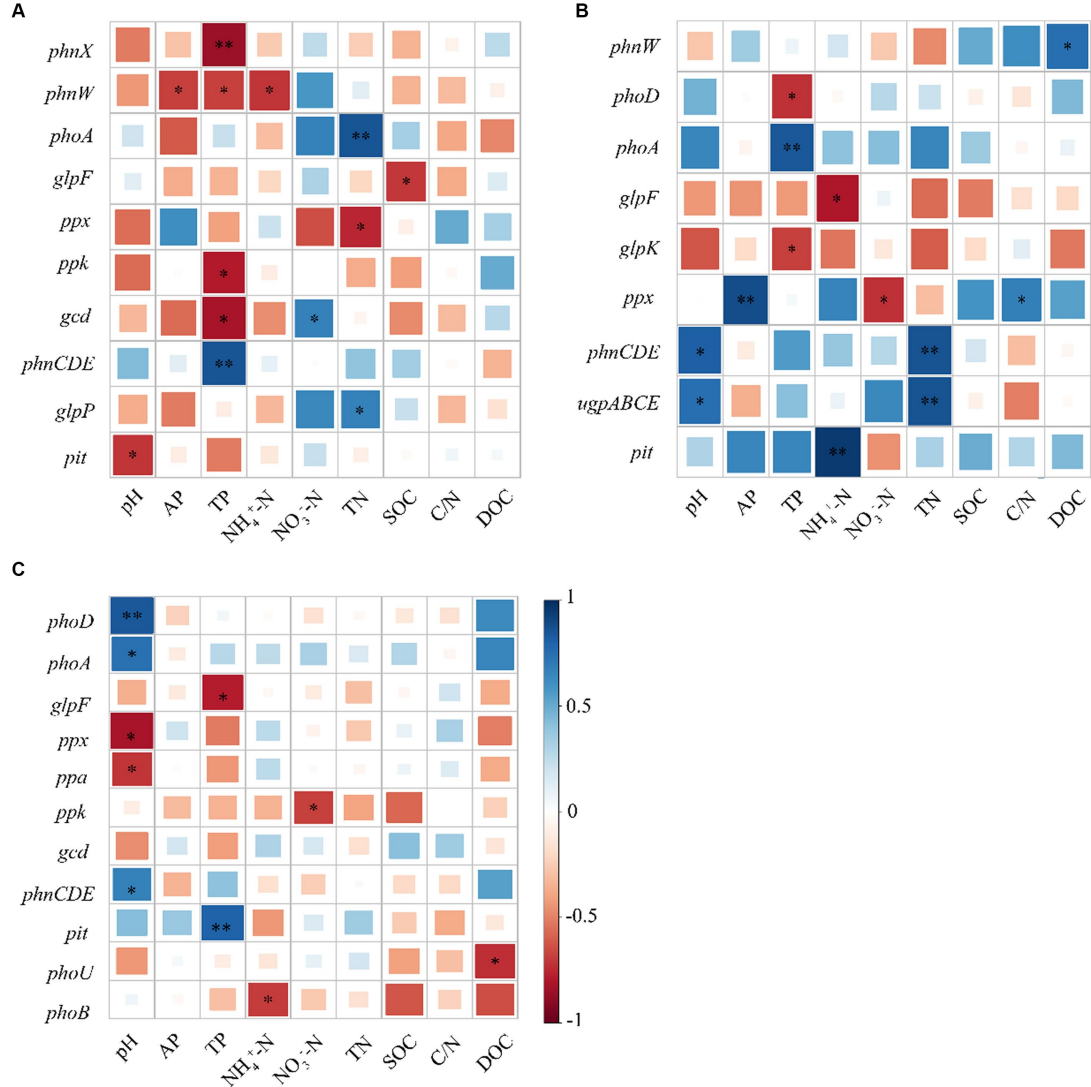
Pearson’s correlation analysis between soil physicochemical properties and functional genes associated with phosphorus cycling; **(A)** 0–20; **(B)** 20–40; and **(C)** 40–60 cm.

As revealed by Pearson’s correlation analysis results, *gcd* showed an obvious negative relationship with TP but a positive relationship with NO_3_^−^-N, whereas *phnW* exhibited a negative relationship with AP, TP, and pH, and likewise, *phnX* was negatively correlated with TP at the topsoil. At the middle layer, *phoD* was negatively correlated with TP. However, there was no correlation between *phoR* and evaluated soil physicochemical properties. It can be seen that almost all genes with significant differences in their relative abundance among the three stands had significant negative correlations with TP (Figure 5).

### 3.4 Molecular ecological network analysis of microbial genes related to P transformation

**Figure 6:**
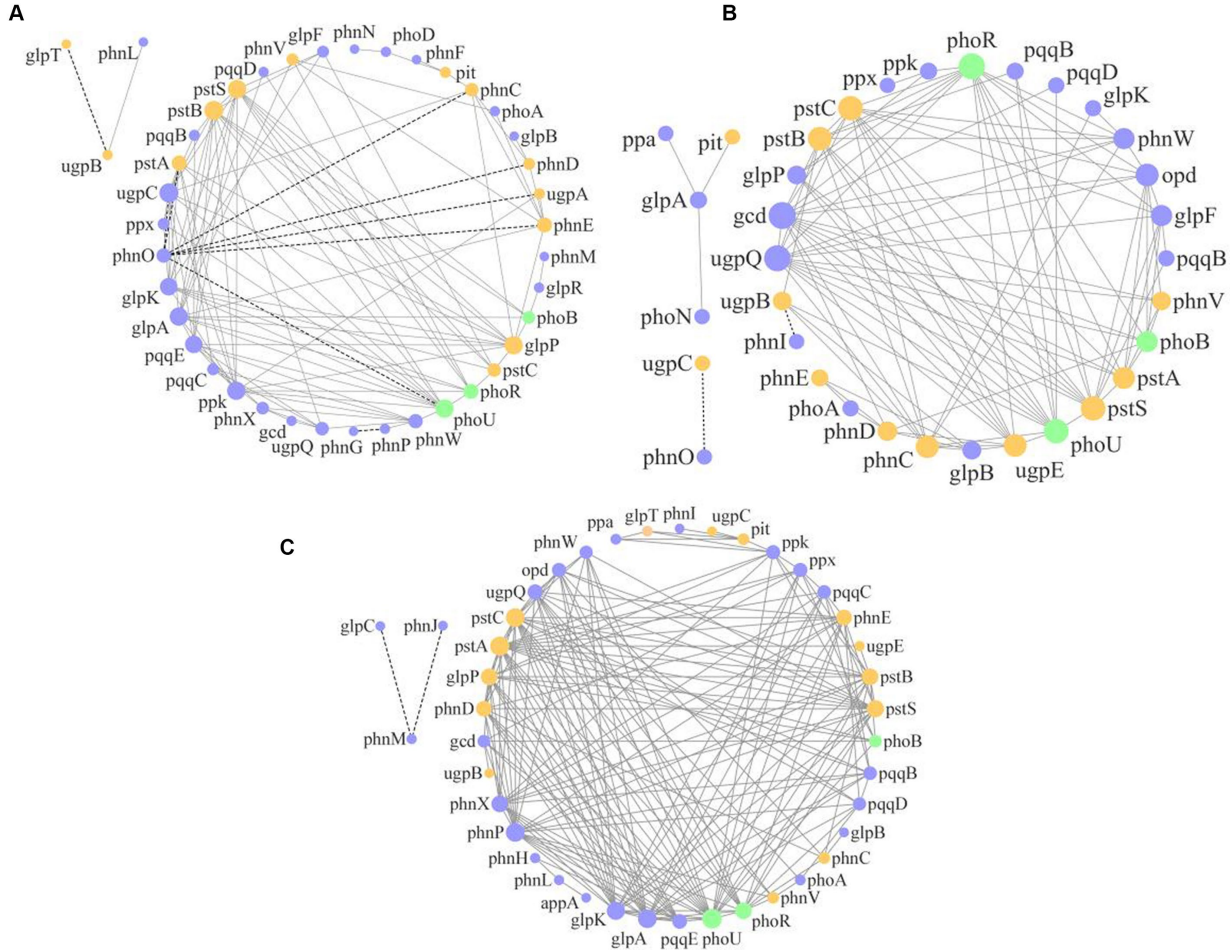
Molecular ecological networks of soil phosphorus cycling microbial functional genes in the PM plantation **(A)**, the EF plantation **(B)**, and the PM/EF plantation **(C)**; Nodes with green, purple, and orange colors represent genes related to P-starvation response regulation, P-solubilization and mineralization, and P-uptake and transport, respectively. The node size indicates the strength of connection with other nodes (genes). Different lines represent the linear correlation between nodes, with solid and dotted lines denoting the positive and negative relations, respectively.

The molecular ecological network analysis of phosphorus cycling microbial functional genes within soil profiles (0–60 cm) under all plantation stands revealed that 41, 34, and 41 gene nodes and 112, 103, and 179 lines were retained for PM, EF, and PM/EF stands, respectively, to construct molecular ecological networks of phosphorus cycling functional genes (Table 5). The connecting lines in three molecular ecological networks represent mainly positive interactions between nodes (Figure 6).

**Table 5:** Topological properties of molecular ecological networks of phosphorus cycling microbial functional genes in three studied stands.

Topological properties	PM	EF	PM/EF
Nodes	41	34	41
Total links	112	103	179
Positive links	103	101	177
Negative links	9	2	2
Mean degree	6.059	5.463	8.732
Mean clustering coefficient	0.420	0.510	0.520
Mean path distance	2.424	2.140	2.864
Modularity	0.281	0.220	0.340

PM, *Pinus massoniana*; EF, *Erythrophleum fordii*.

Topological analysis of molecular ecological networks of soil phosphorus cycling microbial functional genes in 3 studied stands showed that the complexity of the network (average degree), the degree of community clustering (mean clustering coefficient), the distance between nodes (mean path length), and level of community organization (modularity) increased after the transformation of the PM stand into the PM/EF stand. However, after the transformation of the PM plantation stand into the EF stand, only the mean clustering coefficient of the network increased, while mean path length, modularity, and average connectivity decreased.

**Figure 7:**
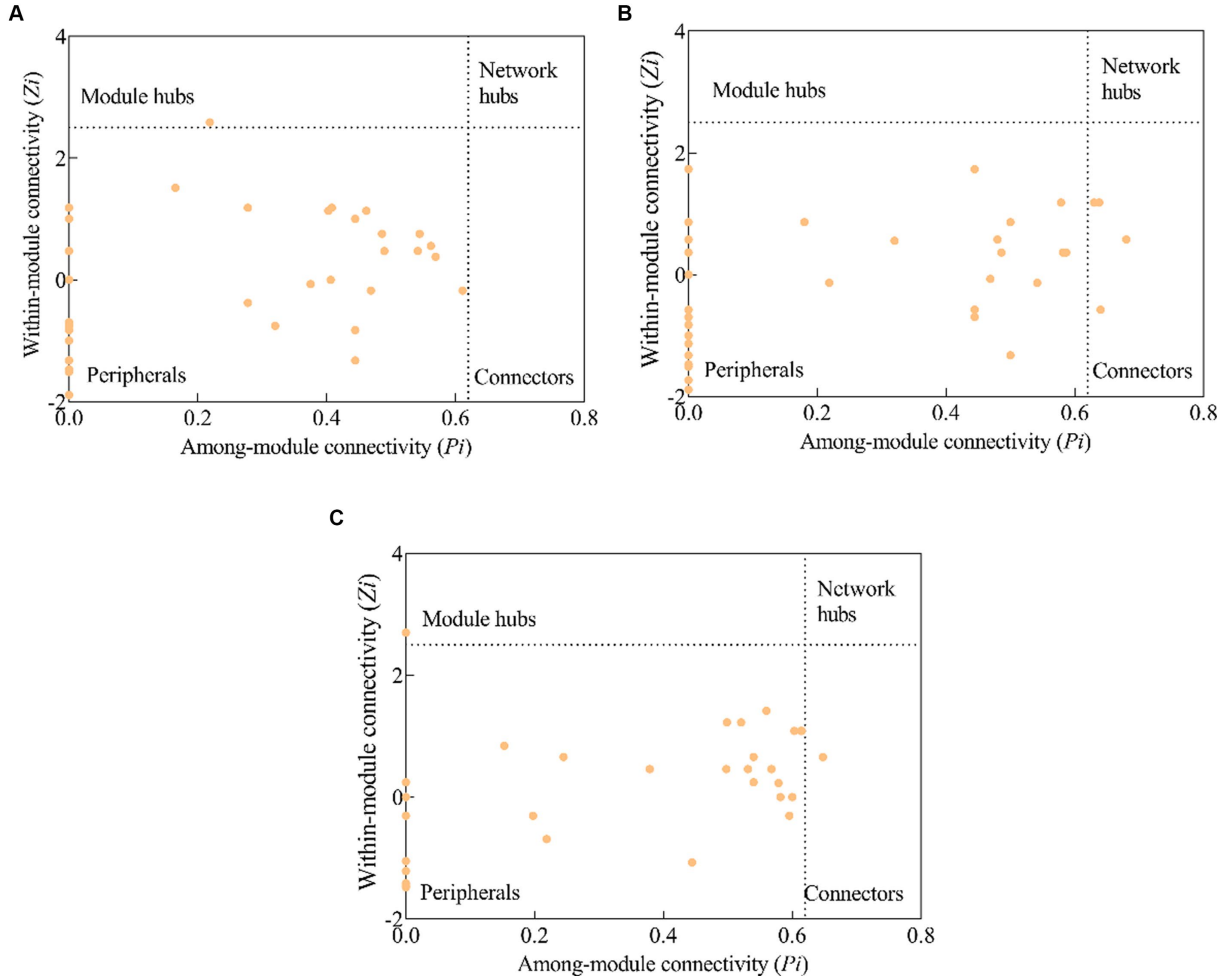
Topological clustering of soil phosphorus cycling microbial functional genes in the PM plantation **(A)**, the EF plantation **(B)**, and the PM/EF plantation **(C)**.

In a molecular ecological network, different genes (nodes) in a microbial community play distinct roles (Fuhrman and Steele, 2008). To determine how plantation conversion affected the topological role of nodes in a network, within- and among-module connectivity (*Zi* and *Pi*, respectively) in three networks were visualized (Figure 7). There was only one module hub (*ugpC*) in the network for the PM plantation, while the network for the EF plantation was found to have four connectors (*phoU*, *pstA*, *gcd*, and *ugpE*), and that of the PM/EF plantation had one module hub (*pqqE*) and one connector (*pstB*); none of the three networks had a network hub (Table 6).

**Table 6:** Module hubs and connecters of the soil phosphorus cycling microbial functional genes molecular ecological networks in different stands.

Stand type	Module hubs		Connectors	
	Species	Classification	Species	Classification
PM	*ugpC*	P-uptake and transportation		
EF			*pstA*, *ugpE*	P-uptake and transportation
			*gcd*	Inorganic P-solubilization
			*phoU*	P-starvation response
PM/EF	*pqqE*	Inorganic P-solubilization	*pstB*	P-uptake and transportation

PM, *Pinus massoniana*; EF, *Erythrophleum fordii*.

## 4 Discussion

### 4.1 Effects of stand types on the composition structure of genes related to P transformation

Differences in the biological characteristics of tree species in forest soils lead to differences in the quantity and quality of litter, thereby affecting soil microbial community structure within the forest floor (Richter et al., 2018). Changes in the composition of microbial functional genes are often associated with alterations in plant and soil characteristics in ecosystems (Hu et al., 2019). Soil TP content is an important factor that affects the composition structure of soil phosphorus cycling microbial functional genes (Yu et al., 2021; Wang et al., 2022). According to our results, there were significant differences among the PM, EF, and PM/EF plantations in terms of the composition structure of phosphorus-cycling microbial functional genes at the topsoil. The main reason for this difference is that the TP content in the PM stand (0.40 g‧kg^−1^) remarkably increased compared to that of the EF stand (0.28 g‧kg^−1^) and the PM/EF stand (0.33 g‧kg^−1^). It is worth noting that in this study, functional gene composition was not significantly different among three stands in the middle layer and subsoil, which indicates that the soil layer classification provides a framework that can deepen our understanding of how stand types affect the composition structure of soil phosphorus cycling microbial functional genes.

### 4.2 Effects of stand types on relative abundances of genes related to P transformation

According to our results, the relative abundance of P-starvation response regulation gene groups in the subsoil significantly decreased in the PM stand compared with that in the EF stand, which is probably attributed to the higher SOC content in this soil layer of the PM stand (16.07 g‧kg^−1^) than that in the EF stand (14.87 g‧kg^−1^). The SOC content showed a negative relationship with the relative abundance of the P-starvation response regulation genes (Li et al., 2022). In addition, concerning the genes involved in inorganic P-solubilization and organic P-mineralization, relative abundances in the topsoil and the middle layer apparently decreased in the PM stand compared to that in the EF stand. Structural equation modeling revealed that stand types could affect soil TP content, which in turn, had a significant negative effect on relative abundances of genes related to inorganic P-solubilization and organic P-mineralization. This is consistent with the results of previous studies indicating that under low-P conditions, genes associated with inorganic P-solubilization and organic P-mineralization enhanced phosphorus availability by encoding phosphatase or releasing organic acids, which can explain the negative correlation of soil TP content with the abundance of these genes (Luo et al., 2020). Additionally, the TP content markedly increased in the topsoil and the middle layer of the PM stand (0.4 and 0.3 g‧kg^−1^, respectively) compared to that of the EF stand (0.28 and 0.23 g‧kg^−1^, respectively), demonstrating it as the main factor leading to the above-mentioned difference.

Dominant functional genes associated with soil phosphorus cycling varied in different stand types (Cheng et al., 2022; Wu et al., 2022). Among the 55 functional genes evaluated in this study, the dominant ones in all soil layers of 3 plantations were *phoR*, *glpP*, *gcd*, *ppk*, and *ppx*, indicating that the dominant functional genes involved in phosphorus cycling in all soil layers (0–60 cm) were not modified after the transformation of the PM plantation into EF and PM/EF plantations. At the same time, the *glpP* gene encoding glycerol-3-phosphate transporter protein was found to have the highest relative abundance, and it can activate the expression of genes related to the mineralization and transport of glycerol-3-phosphate such as *glpF*, *glpK*, *glpT*, etc. (Darbon et al., 2002). This indicated that the microorganisms in the soil under the three plantations could absorb glycerol-3-phosphate as an alternative phosphorus source to maintain the basic functions of cells, thus coping with phosphorus starvation (Bergkemper et al., 2016).

Microbial solubilization of inorganic phosphorus is mainly regulated by the *gcd* gene encoding quinoprotein glucose dehydrogenase, which is an enzyme that secretes gluconic acid that chelates Ca^2+^, Fe^3+^, and Al^3+^ cations, thus dissolving mineral phosphorus that is not directly available to plants and organisms in the soil and releasing available phosphorus (Zeng et al., 2016). The *gcd* gene not only is ubiquitous in soil but also can be used as a key molecular marker to characterize available phosphorus in soil (Rawat et al., 2020). In this study, *gcd* in each soil layer under the three stands was dominant, indicating that the microbial solubilization of inorganic phosphorus was the main process providing soil available phosphorus for stands (Liang et al., 2020). At 0–20 cm, *gcd* was significantly negatively correlated with TP content, which in the PM plantation (0.4 g‧kg^−1^) apparently increased compared with that in the EF plantation (0.28 g‧kg^−1^). Therefore, soil TP is one of the most important factors that significantly increased the relative abundance of *gcd* after the transformation of the PM plantation into the EF plantation. Previous studies have also pointed out that it is easier for microorganisms to obtain nutrients from the soil with a higher TP content, which leads to a lower investment of microorganisms in the process of phosphorus solubilization, and thus a decrease in relative abundances of related functional genes (Pastore et al., 2020). In addition, the increase in the NO_3_^−^-N content would increase the demand of microorganisms for inorganic phosphorus, resulting in the improvement of the capacity of microbes to dissolve phosphorus to obtain more inorganic phosphorus for their survival (Xiao et al., 2018). This study found that the relative abundance of the *gcd* gene was significantly positively correlated with the NO_3_^−^-N content. The NO_3_^−^-N content in the 0–20 cm of the PM plantation was significantly lower (0.48 mg‧kg^−1^) than that of the EF plantation (1.56 mg‧kg^−1^), which is another important reason for the higher relative abundance of *gcd* in the latter.

### 4.3 Effects of stand types on molecular ecological network structure of key genes involved in P transformation

There are many microorganisms associated with complex P-cycling processes in soil (Billah et al., 2019), and molecular ecological networks can reveal the potential interactions of microorganisms (Deng et al., 2012). Compared to simpler networks, in complex networks, microbial communities have more efficient resource utilization and information transmission (Morrien et al., 2017). In this study, the average degree of molecular ecological networks of soil phosphorus cycling microbial functional genes was higher in the PM stand than in the EF stand, but lower than that in the PM/EF stand, indicating that the network complexity was reduced after the transformation of the PM plantation stand into the EF stand but it increased after the transformation into the PM/EF stand. This is consistent with previous research results, reporting that compared with pure plantations, the molecular ecological network structure of phosphorus cycling functional genes was more complex in the mixed plantation (Cheng et al., 2021). Furthermore, the diversity of tree species, litter, and root exudates in mixed forests was generally higher than in pure forests, which may lead to more complex interactions between soil microorganisms (Haichar et al., 2014).

The mean clustering coefficient of molecular networks of soil phosphorus cycling microbial functional genes in the PM stand was lower than that in EF and PM/EF stands, indicating that the level of community organization was the lowest in the PM stand. The lower value of mean path length indicates the closer interaction between genes within this network and the faster diffusion of external interference in networks, resulting in the instability of the network structure (Deng et al., 2012). Higher modularity values indicate a stronger anti-interference ability (Carpenter et al., 2012). The average path length and modularity of the network of the PM stand increased compared to those of the EF stand but they had lower values than those of the PM/EF stand, indicating that soil phosphorus cycling microbial functional genes under this stand type could be more closely functionally related and be more tolerant to environmental disturbances (Yuan et al., 2021), while in EF, the microbial functional genes of soil phosphorus cycling were susceptible to the interference of external environment, and more key genes were needed to maintain the stability of molecular ecological network (Banerjee et al., 2018). In addition, the functional gene networks for the three stands had mainly positive connections, indicating that microbial functional genes involved in soil phosphorus cycling may obtain limited nutrient sources through cooperation (Hoek et al., 2016).

Module hubs and connectors are key genes in functional molecular ecological networks, and they are also critical for maintaining the stability of community structure (Banerjee et al., 2018). In the present study, *ugpC* was identified as the key gene in the PM plantation, whereas *phoU*, *pstA*, *ugpE*, and *gcd* were the key genes in the EF plantation, and *pqqE* and *pstB* were the key genes in the PM/EF plantation, all associated with different P cycling processes. Numerous recent studies demonstrate the potential keystone roles of pyrroloquinoline quinone (*pqq*) and glucose dehydrogenase (*gcd*) in phosphorus solubilization, and thus, they are becoming reference biomarkers in research on soil P cycling (Rawat et al., 2020). The *pstA*, *pstB*, *ugpC*, and *ugpE* genes were expressed by pst and ugp transport systems (*pstSCAB* and *ugpABE*, respectively) (Dai et al., 2020). One study has shown that *pstB* and *ugpA* are the key functional genes related to soil P cycling (Liu et al., 2023), which conforms to our findings. However, to further verify our results, it should be explored whether the gene coding for the negative regulatory protein *phoU* plays a key role in P-cycling processes. The modification of key functional genes occurred by the transformation of the PM plantation into EF and PM/EF plantations, which may be due to its influence on the soil microenvironment, thus modulating the response mechanism of microorganisms to environmental disturbances.

Overall, this study only collected soil samples in winter, which could not characterize the effects of seasonal changes on the microbial functional genes of soil phosphorus cycling. Soil samples can be collected in different seasons to elucidate the effects of different stand types on soil phosphorus cycling in future.

## 5 Conclusion

The composition structure of soil phosphorus cycling microbial functional genes in the PM plantation was significantly different from that in EF and PM/EF plantations in the subtropics of China, mainly due to the difference in soil TP content. After the transformation of the PM plantation stand into the EF stand, the relative abundances of the group of genes involved in inorganic P-solubilization and organic P-mineralization in the topsoil and middle layer significantly increased, and the difference was caused by TP. At the same time, for genes related to P-starvation response regulation, relative abundances in the subsoil increased, and SOC may be the main factor contributing to this difference. The transformation of the PM stand into PM/EF and EF plantation stands had no obvious influence on the dominant phosphorus cycling microbial functional genes in all soil layers of stands. The decrease in the TP content and the increase in the NO_3_^−^-N content in the topsoil after the transformation of the PM stand into the EF stand are the reasons for the significant increase in the relative abundance of *gcd* in the EF stand. After the conversion of the PM plantation into the PM/EF plantation, the molecular ecological network structure of soil phosphorus cycling microbial functional genes became more complex, and thus, its stability was enhanced. Moreover, the key genes that play roles in maintaining network stability in different stands exhibited an inconsistent pattern of change. Future studies may be required to focus on the processes of gene transcription and translation, which will help to more comprehensively understand how plantation transformation affects the expression of soil phosphorus cycling microbial functional genes and its potential mechanism.

## Data availability statement

The sequencing data have been deposited in the National Microbiology Data Center (https://nmdc.cn) under NMDC10018441.

## Ethics statement

The manuscript presents research on animals that do not require ethical approval for their study.

## Author contributions

LQ: Conceptualization, Funding acquisition, Investigation, Methodology, Project administration, Writing – original draft, Writing – review & editing. ZX: Conceptualization, Formal analysis, Investigation, Methodology, Writing – original draft, Writing – review & editing. AM: Funding acquisition, Investigation, Writing – review & editing. JT: Formal analysis, Methodology, Writing – original draft. HZ: Investigation, Writing – original draft. JQ: Investigation, Writing – original draft. ZL: Investigation, Writing – original draft.

## Footnotes

^1^ https://nmdc.cn

^2^ http://blast.ncbi.nlm.nih.gov/Blast.cgi

^3^ http://www.genome.jp/kegg/

^4^ http://ieg4.rccc.ou.edu/mena/

^5^ http://manual.cytoscape.org/en/stable/

## References

[ref1] An R. Moe L. A. (2016). Regulation of pyrroloquinoline quinone-dependent glucose dehydrogenase activity in the model rhizosphere-dwelling bacterium *Pseudomonas putida* KT2440. Appl. Environ. Microbiol. 82, 4955–4964. doi: 10.1128/AEM.00813-16, PMID: 27287323 PMC4968544

[ref2] Andreeva I. G. Golubeva L. I. Kuvaeva T. M. Gak E. R. Katashkina J. I. Mashko S. V. (2011). Identification of *Pantoea ananatis* gene encoding membrane pyrroloquinoline quinone (PQQ)-dependent glucose dehydrogenase and pqqABCDEF operon essential for PQQ biosynthesis. FEMS Microbiol. Lett. 318, 55–60. doi: 10.1111/j.1574-6968.2011.02240.x, PMID: 21306430

[ref3] Banerjee S. Schlaeppi K. van der Heijden M. G. A. (2018). Keystone taxa as drivers of microbiome structure and functioning. Nat. Rev. Microbiol. 16, 567–576. doi: 10.1038/s41579-018-0024-1, PMID: 29789680

[ref4] Bao S. (2000). Analytical methods for soil and agro-chemistry. Beijing: China Agricultural Press.

[ref5] Bardgett R. D. van der Putten W. H. (2014). Belowground biodiversity and ecosystem functioning. Nature 515, 505–511. doi: 10.1038/nature13855 25428498

[ref6] Becerra A. Bartoloni N. Cofré N. Soteras F. Cabello M. (2014). Arbuscular mycorrhizal fungi in saline soils: vertical distribution at different soil depth. Braz. J. Microbiol. 45, 585–594. doi: 10.1590/S1517-83822014000200029 25242945 PMC4166286

[ref7] Beijer L. Rutberg L. (1992). Utilisation of glycerol and glycerol 3-phosphate is differently affected by the phosphotransferase system in *Bacillus subtilis*. FEMS Microbiol. Lett. 100, 217–220. doi: 10.1111/j.1574-6968.1992.tb14043.x, PMID: 1335945

[ref8] Bergkemper F. Schöler A. Engel M. Lang F. Krüger J. Schloter M.. (2016). Phosphorus depletion in forest soils shapes bacterial communities towards phosphorus recycling systems. Environ. Microbiol. 18, 1988–2000. doi: 10.1111/1462-2920.13188, PMID: 26690731

[ref9] Billah M. Khan M. Bano A. Hassan T. U. Munir A. Gurmani A. R. (2019). Phosphorus and phosphate solubilizing bacteria: keys for sustainable agriculture. Geomicrobiol J. 36, 904–916. doi: 10.1080/01490451.2019.1654043

[ref10] Cade-Menun B. J. Bainard L. D. LaForge K. Schellenberg M. Houston B. Hamel C. (2017). Long-term agricultural land use affects chemical and physical properties of soils from Southwest Saskatchewan. Can. J. Soil Sci. 97, 650–666. doi: 10.1139/CJSS-2016-0153

[ref11] Carpenter S. R. Arrow K. J. Barrett S. Biggs R. Brock W. A. Crépin A.. (2012). General resilience to cope with extreme events. Sustain. For. 4, 3248–3259. doi: 10.3390/su4123248

[ref12] Cheng J. Han Z. Cong J. Yu J. Zhou J. Zhao M.. (2021). Edaphic variables are better indicators of soil microbial functional structure than plant-related ones in subtropical broad-leaved forests. Sci. Total Environ. 773:145630. doi: 10.1016/j.scitotenv.2021.145630, PMID: 33582323

[ref13] Cheng J. Zhang Y. Wang H. Cui Z. Cao C. (2022). Sand-fixation plantation type affects soil phosphorus transformation microbial community in a revegetation area of Horqin Sandy land, Northeast China. Ecol. Eng. 180:106644. doi: 10.1016/j.ecoleng.2022.106644

[ref14] Choi E. Groisman E. A. Shin D. (2009). Activated by different signals, the PhoP/PhoQ two-component system differentially regulates metal uptake. J. Bacteriol. 191, 7174–7181. doi: 10.1128/JB.00958-09, PMID: 19801407 PMC2786564

[ref15] Dai Z. Liu G. Chen H. Chen C. Wang J. Ai S.. (2020). Long-term nutrient inputs shift soil microbial functional profiles of phosphorus cycling in diverse agroecosystems. ISME J. 14, 757–770. doi: 10.1038/s41396-019-0567-9, PMID: 31827246 PMC7031380

[ref16] Darbon E. Servant P. Poncet S. Deutscher J. (2002). Antitermination by GlpP, catabolite repression via CcpA and inducer exclusion triggered by P-GlpK dephosphorylation control *Bacillus subtilis glpFK* expression. Mol. Microbiol. 43, 1039–1052. doi: 10.1046/j.1365-2958.2002.02800.x, PMID: 11929549

[ref17] Deng Y. Jiang Y. Yang Y. He Z. Luo F. Zhou J. (2012). Molecular ecological network analyses. BMC Bioinform. 13:113. doi: 10.1186/1471-2105-13-113, PMID: 22646978 PMC3428680

[ref18] Dixon P. (2003). VEGAN, a package of R functions for community ecology. J. Veg. Sci. 14, 927–930. doi: 10.1111/j.1654-1103.2003.tb02228.x

[ref9001] Fuhrman J. A. Steele J. A. (2008). Community structure of marine bacterioplankton: patterns, networks, and relationships to function. Aquat Microb Ecol 53, 69–81. doi: 10.3354/ame01222, PMID: 20171928

[ref19] Gillespie L. M. Hättenschwiler S. Milcu A. Wambsganss J. Shihan A. Fromin N. (2021). Tree species mixing affects soil microbial functioning indirectly via root and litter traits and soil parameters in European forests. Funct. Ecol. 35, 2190–2204. doi: 10.1111/1365-2435.13877

[ref20] Haichar F. Z. Santaella C. Heulin T. Achouak W. (2014). Root exudates mediated interactions belowground. Soil Biol. Biochem. 77, 69–80. doi: 10.1016/j.soilbio.2014.06.017

[ref21] Handa I. T. Aerts R. Berendse F. Berg M. P. Bruder A. Butenschoen O.. (2014). Consequences of biodiversity loss for litter decomposition across biomes. Nature 509, 218–221. doi: 10.1038/nature13247, PMID: 24805346

[ref22] He Y. Qin L. Li Z. Liang X. Shao M. Tan L. (2013). Carbon storage capacity of monoculture and mixed-species plantations in subtropical China. For. Ecol. Manag. 295, 193–198. doi: 10.1016/j.foreco.2013.01.020

[ref23] Hoek T. A. Axelrod K. Biancalani T. Yurtsev E. A. Liu J. Gore J. (2016). Resource availability modulates the cooperative and competitive nature of a microbial cross-feeding mutualism. PLoS Biol. 14:e1002540. doi: 10.1371/journal.pbio.1002540, PMID: 27557335 PMC4996419

[ref24] Hooper D. Coughlan J. Mullen M. (2008). Structural equation modelling: guidelines for determining model fit. Electron. J. Bus. Res. Methods 6, 53–60.

[ref25] Hsieh Y. Wanner B. (2010). Global regulation by the seven-component pi signaling system. Curr. Opin. Microbiol. 13, 198–203. doi: 10.1016/j.mib.2010.01.014, PMID: 20171928 PMC2847643

[ref26] Hu M. Peñuelas J. Sardans J. Tong C. Chang C. T. Cao W. (2020). Dynamics of phosphorus speciation and the *phoD* phosphatase gene community in the rhizosphere and bulk soil along an estuarine freshwater-oligohaline gradient. Geoderma 365:114236. doi: 10.1016/j.geoderma.2020.114236

[ref27] Hu Y. Zhang Z. Huang L. Qi Q. Liu L. Zhao Y.. (2019). Shifts in soil microbial community functional gene structure across a 61-year desert revegetation chronosequence. Geoderma 347, 126–134. doi: 10.1016/j.geoderma.2019.03.046

[ref28] Jiang W. Metcalf W. W. Lee K. S. Wanner B. L. (1995). Molecular cloning, mapping, and regulation of pho regulon genes for phosphonate breakdown by the phosphonatase Pathway of *Salmonella typhimurium* LT2. J. Bacteriol. 177, 6411–6421. doi: 10.1128/jb.177.22.6411-6421.1995, PMID: 7592415 PMC177490

[ref29] Kelliher J. L. Radin J. N. Kehl-Fie T. E. (2018). PhoPR contributes to *Staphylococcus aureus* during phosphate starvation and pathogenesis in an environment-specific manner. Infect. Immun. 86, e00371–18. doi: 10.1128/IAI.00371-18, PMID: 30061377 PMC6204748

[ref30] Li Y. Wang J. He L. Xu X. Wang J. Ren C.. (2022). Different mechanisms driving increasing abundance of microbial phosphorus cycling gene groups along an elevational gradient. iScience 25:105170. doi: 10.1016/j.isci.2022.105170, PMID: 36204265 PMC9529982

[ref31] Liang J. Liu J. Jia P. Yang T. Zeng Q. Zhang S.. (2020). Novel phosphate-solubilizing bacteria enhance soil phosphorus cycling following ecological restoration of land degraded by mining. ISME J. 14, 1600–1613. doi: 10.1038/s41396-020-0632-4, PMID: 32203124 PMC7242446

[ref32] Liang Y. Zhao H. Deng Y. Zhou J. Li G. Sun B. (2016). Long-term oil contamination alters the molecular ecological networks of soil microbial functional genes. Front. Microbiol. 7:60. doi: 10.3389/fmicb.2016.00060 26870020 PMC4737900

[ref33] Liu J. Cade-Menun B. J. Yang J. Hu Y. Liu C. W. Tremblay J.. (2018). Long-term land use affects phosphorus speciation and the composition of phosphorus cycling genes in agricultural soils. Front. Microbiol. 9:1643. doi: 10.3389/fmicb.2018.01643, PMID: 30083148 PMC6065304

[ref34] Liu L. Gao Z. Yang Y. Gao Y. Mahmood M. Jiao H.. (2023). Long-term high-P fertilizer input shifts soil P cycle genes and microorganism communities in dryland wheat production systems. Agric. Ecosyst. Environ. 342:108226. doi: 10.1016/j.agee.2022.108226

[ref35] Liu S. Yang Y. Wang H. (2018). Development strategy and management countermeasures of planted forests in China: transforming from timber-centered single objective management towards multi-purpose management for enhancing quality and benefits of ecosystem services. Acta Ecol. Sin. 38, 1–10. doi: 10.5846/stxb201712072201

[ref36] Lucero C. T. Lorda G. S. Anzuay M. S. Luduena L. M. Taurian T. (2021). Peanut endophytic phosphate solubilizing bacteria increase growth and P content of soybean and maize plants. Curr. Microbiol. 78, 1961–1972. doi: 10.1007/s00284-021-02469-x, PMID: 33839883

[ref37] Luo D. Shi Z. Tang J. Liu S. Lu L. (2014). Soil microbial community structure of monoculture and mixed plantation stands of native tree species in south subtropical China. Chin. J. Appl. Ecol. 25, 2543–2550. doi: 10.13287/j.1001-9332.20140611.001, PMID: 25757303

[ref38] Luo G. Xue C. Jiang Q. Xiao Y. Zhang F. Guo S.. (2020). Soil carbon, nitrogen, and phosphorus cycling microbial populations and their resistance to global change depend on soil C: N: P stoichiometry. mSystems 5, e00162–e00120. doi: 10.1128/mSystems.00162-20 32606023 PMC7329320

[ref39] Morrien E. Hannula S. E. Snoek L. B. Helmsing N. R. Zweers H. de Hollander M.. (2017). Soil networks become more connected and take up more carbon as nature restoration progresses. Nat. Commun. 8:14349. doi: 10.1038/ncomms14349, PMID: 28176768 PMC5309817

[ref40] Olesen J. M. Bascompte J. Dupon Y. L. Jordano P. (2007). The modularity of pollination networks. Proc. Natl. Acad. Sci. U. S. A. 104, 19891–19896. doi: 10.1073/pnas.0706375104 18056808 PMC2148393

[ref41] Pastore G. Kernchen S. Spohn M. (2020). Microbial solubilization of silicon and phosphorus from bedrock in relation to abundance of phosphorus-solubilizing bacteria in temperate forest soils. Soil Biol. Biochem. 151:108050. doi: 10.1016/j.soilbio.2020.108050

[ref42] Rawat P. Das S. Shankhdhar D. Shankhdhar S. C. (2020). Phosphate-solubilizing microorganisms: mechanism and their role in phosphate solubilization and uptake. J. Soil Sci. Plant Nutr. 21, 49–68. doi: 10.1007/s42729-020-00342-7

[ref43] Reinhard C. T. Planavsky N. J. Gill B. C. Ozaki K. Robbins L. J. Lyons T. W.. (2017). Evolution of the global phosphorus cycle. Nature 541, 386–389. doi: 10.1038/nature20772 28002400

[ref44] Richter A. Schöning I. Kahl T. Bauhus J. Ruess L. (2018). Regional environmental conditions shape microbial community structure stronger than local forest management intensity. For. Ecol. Manag. 409, 250–259. doi: 10.1016/j.foreco.2017.11.027

[ref45] Schall P. Ammer C. (2012). How to quantify forest management intensity in central European forests. Eur. J. For. Res. 132:397. doi: 10.1007/s10342-012-0659-9

[ref46] Siles J. A. Starke R. Martinovic T. Fernandes M. L. P. Orgiazzi A. Bastida F. (2022). Distribution of phosphorus cycling genes across land uses and microbial taxonomic groups based on metagenome and genome mining. Soil Biol. Biochem. 174:108826. doi: 10.1016/j.soilbio.2022.108826

[ref47] Tan H. Barret M. Mooij M. Rice O. Morrissey J. P. Dobson A.. (2012). Long-term phosphorus fertilisation increased the diversity of the total bacterial community and the *phoD* phosphorus mineraliser group in pasture soils. Biol. Fertil. Soils 49, 661–672. doi: 10.1007/s00374-012-0755-5

[ref48] Wang H. Liu S. Wang J. You Y. Yang Y. Shi Z.. (2018). Mixed-species plantation with *Pinus massoniana* and *Castanopsis hystrix* accelerates C loss in recalcitrant coniferous litter but slows C loss in labile broadleaf litter in southern China. For. Ecol. Manag. 422, 207–213. doi: 10.1016/j.foreco.2018.04.024

[ref49] Wang L. Wen Y. Tong R. Zhang H. Chen H. Hu T.. (2022). Understanding responses of soil microbiome to the nitrogen and phosphorus addition in *Metasequoia glyptostroboides* plantations of different ages. Microb. Ecol. 84, 565–579. doi: 10.1007/s00248-021-01863-z, PMID: 34545413

[ref50] Wang F. Zhang Y. Xia Y. Cui Z. Cao C. (2021). Soil microbial community succession based on *PhoD* and *Gcd* genes along a chronosequence of sand-fixation forest. Forests 12, 1707. doi: 10.3390/f12121707

[ref51] Wu H. Du S. Zhang Y. An J. Zou H. Zhang Y.. (2019). Effects of irrigation and nitrogen fertilization on greenhouse soil organic nitrogen fractions and soil-soluble nitrogen pools. Agric. Water Manag. 216, 415–424. doi: 10.1016/j.agwat.2019.02.020

[ref52] Wu X. Rensing C. Han D. Xiao K. Dai Y. Tang Z.. (2022). Genome-resolved metagenomics reveals distinct phosphorus acquisition strategies between soil microbiomes. mSystems 7, e01107–e01121. doi: 10.1128/msystems.01107-21 35014868 PMC8751388

[ref53] Xiao W. Chen X. Jing X. Zhu B. (2018). A meta-analysis of soil extracellular enzyme activities in response to global change. Soil Biol. Biochem. 123, 21–32. doi: 10.1016/j.soilbio.2018.05.001

[ref54] Xie C. Mao X. Huang J. (2011). KOBAS 2.0: a web server for annotation and identification of enriched pathways and diseases. Nucleic Acids Res. 39, W316–W322. doi: 10.1093/nar/gkr483, PMID: 21715386 PMC3125809

[ref55] You Y. Huang X. Zhu H. Liu S. Liang H. Wen Y.. (2018b). Positive interactions between *Pinus massoniana* and *Castanopsis hystrix* species in the uneven-aged mixed plantations can produce more ecosystem carbon in subtropical China. For. Ecol. Manag. 410, 193–200. doi: 10.1016/j.foreco.2017.08.025

[ref56] You Y. Wu X. Ming A. Liu T. Chen Y. Zhu H.. (2018a). Changes of plant functional group in understory and environmental interpretation in the transformation of typical coniferous plantation to native broadleaved species plantation in south subtropical China. Chin. J. Ecol. 37, 3194–3201. doi: 10.13292/j.1000-4890.201811.025

[ref57] You Y. Xu H. Wu X. Zhou X. Tan X. Li M.. (2020). Native broadleaf tree species stimulate topsoil nutrient transformation by changing microbial community composition and physiological function, but not biomass in subtropical plantations with low P status. For. Ecol. Manag. 477:118491. doi: 10.1016/j.foreco.2020.118491

[ref58] Yu H. Wang F. Shao M. Huang L. Xie Y. Xu Y.. (2021). Effects of rotations with legume on soil functional microbial communities involved in phosphorus transformation. Front. Microbiol. 12:661100. doi: 10.3389/fmicb.2021.661100, PMID: 34659135 PMC8519609

[ref59] Yuan M. M. Guo X. Wu L. Zhang Y. Xiao N. Ning D.. (2021). Climate warming enhances microbial network complexity and stability. Nat. Clim. Chang. 11, 343–348. doi: 10.1038/s41558-021-00989-9

[ref60] Zaidi A. Khan M. S. Ahemad M. Oves M. (2009). Plant growth promotion by phosphate solubilizing bacteria. Acta Microbiol. Immunol. Hung. 56, 263–284. doi: 10.1556/AMicr.56.2009.3.6 19789141

[ref61] Zeng Q. Wu X. Wen X. (2016). Effects of soluble phosphate on phosphate-solubilizing characteristics and expression of *gcd* gene in *Pseudomonas frederiksbergensis* JW-SD2. Curr. Microbiol. 72, 198–206. doi: 10.1007/s00284-015-0938-z, PMID: 26573634

[ref62] Zhou J. Deng Y. Luo F. He Z. Tu Q. Zhi X. (2010). Functional molecular ecological networks. MBio 1, e00169–e00110. doi: 10.1128/mBio.00169-10 20941329 PMC2953006

